# Bronchoplasty for pulmonary preservation: A novel technique

**DOI:** 10.1016/j.xjtc.2023.03.010

**Published:** 2023-03-28

**Authors:** M. Blair Marshall, Evert A. Sugarbaker

**Affiliations:** a Division of Thoracic Surgery, Brigham and Women's Hospital, Boston, Mass; b Harvard Medical School, Boston, Mass

**Figure:**
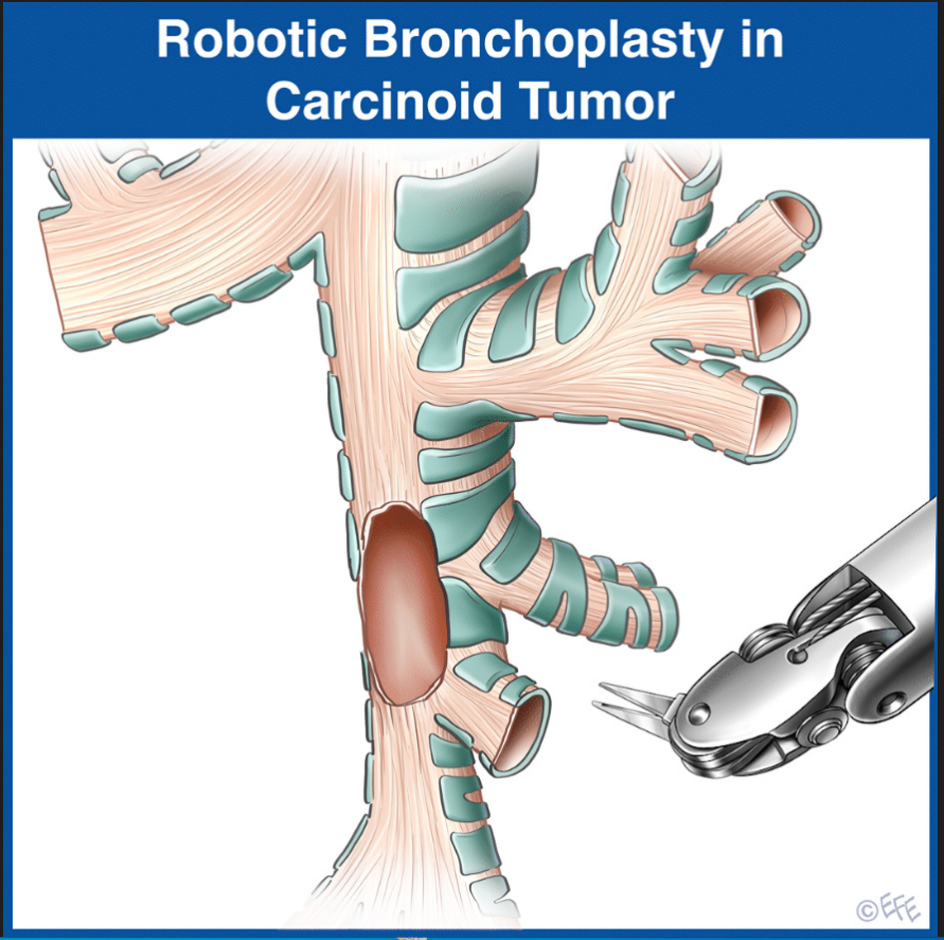
Animation of novel bronchoplasty technique.

**Central Message**

Preservation of uninvolved tissue during resection of low-grade airway tumors can allow for novel reconstruction techniques that preserve pulmonary function.

Bronchoplasty is a technique used to preserve pulmonary function in patients with central airway tumors. This typically incorporates a “sleeve” technique or circumferential resection of the airway with primary reconstruction of the remaining uninvolved bronchus and lung. This technique was an advance in thoracic surgery for the management of central airway tumors allowing preservation of pulmonary function and avoidance of a pneumonectomy. More recently, minimally invasive techniques, both video-assisted thoracic surgery and robotics, have been increasingly utilized when performing these major operations.^1^ In particular, the robotic technology allows for improved visualization through a binocular camera with greater magnification. The wristed instrumentation allows greater precision for cutting and sewing when using a minimally invasive technique. Because of these improvements, the options for resection and reconstruction with minimally invasive techniques has grown.^2^

Endoscopic bronchoscopic techniques have also evolved; however, without complete resection, local recurrence can occur.^3^ The surgical management of low-grade tumors requires local resection with negative margins and a creative approach to bronchoplasty may be of use. For these patients, an atypical approach to resection and reconstruction may be more appropriate. We present a case where a novel approach allowed for preservation of pulmonary function.

A 58-year-old healthy avid cyclist with a carcinoid tumor of the right lower lobe presented initially at age 50 years (Figure 1, *A*). He underwent endoscopic bronchoscopic treatment, including laser. Over time, the extrabronchial component of the tumor continued to grow (Video 1). He ultimately sought our opinion. He underwent robotic resection of the posterior membranous portion of the right lower lobe bronchus (Figure 1, *B* and *C*). The superior segmental bronchus was then used as a flap for reconstruction avoiding a middle and lower lobectomy (Video 2). The superior segmentectomy was completed with a pericardial fat pad-thymic flap. He was discharged without complications on postoperative day 2. Pathology revealed typical carcinoid; margins and nodes were negative. He is well without recurrence at 2.5 years’ follow-up (Video 3). A comprehensive video demonstrating the entire case may be viewed here (Video 4).

**Figure 1:**
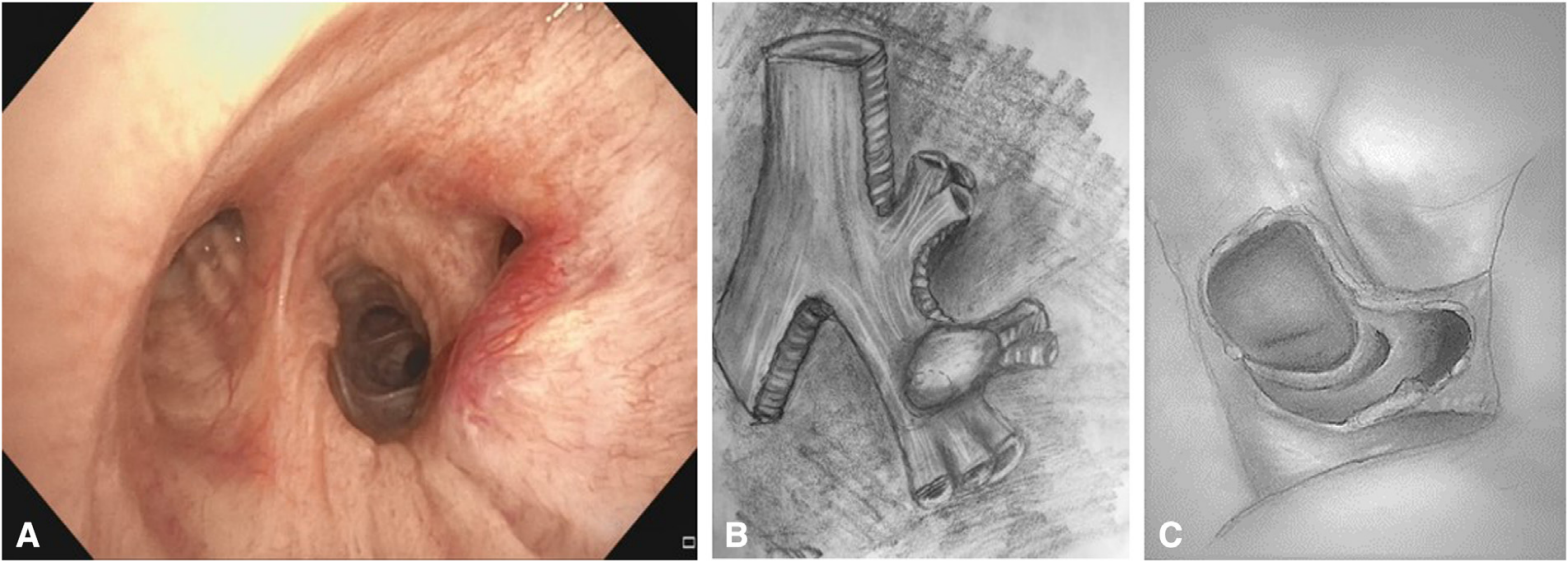
A, Bronchoscopic view of recurrent tumor involving the *lower* lobe bronchus opposite the *middle* lobe bronchus (B) illustration demonstrating location of tumor on the posterior *right lower* lobe bronchus (*dotted line*). C, Illustration demonstrating the postresection defect.

This case demonstrates a novel approach to bronchoplasty. When performed open or minimally invasively, it can allow for maximization of pulmonary preservation in appropriate patients. When contemplating management of airway tumors multiple factors need to be taken into consideration. The ideal management strategy may include a variety of types of resection and reconstruction that are very much specific to each case. However, maximization of pulmonary function should be taken into consideration when devising a reconstructive strategy. When circumferential sleeve resection is the correct choice, it is an excellent option. We and others have reported circumferential sleeve resection for benign stricture or adenoma of the bronchus intermedius.^4^^,^^5^ In this setting, circumferential sleeve is the ideal resection strategy because no parenchyma was removed in these patients. In this patient, because of the location of the tumor, this strategy would not be suitable.

We stress contemplating all possible technical strategies because they are not always entertained. Key surgical principles should be followed, including complete resection of the pathology with negative margins. Plastic principles of preservation of all healthy tissue—while tissue transfer options are considered—may allow for maximum preservation of pulmonary function in some patients.

## Supplementary Data

Video 1 Preoperative axial and sagittal computed tomography images demonstrating the location of the tumor. Video available at: https://www.jtcvs.org/article/S2666-2507(23)00108-6/fulltext.

Video 2 Animation of plasty technique used for resection and reconstruction. Video available at: https://www.jtcvs.org/article/S2666-2507(23)00108-6/fulltext.

Video 3 Postoperative surveillance axial computed tomography images demonstrating widely patent reconstruction and pericardial fat pad-thymic flap coursing through fissure. Video available at: https://www.jtcvs.org/article/S2666-2507(23)00108-6/fulltext.

Video 4 Comprehensive case video with voice over corresponding description. Video available at: https://www.jtcvs.org/article/S2666-2507(23)00108-6/fulltext.
