# Supplementary figures and images for: Bronchoplasty for pulmonary preservation: A novel technique
Source: JTCVS Tech. 2023 Mar 28;19:132–4. doi: 10.1016/j.xjtc.2023.03.010 (PMC10267971; doi:10.1016/j.xjtc.2023.03.010)

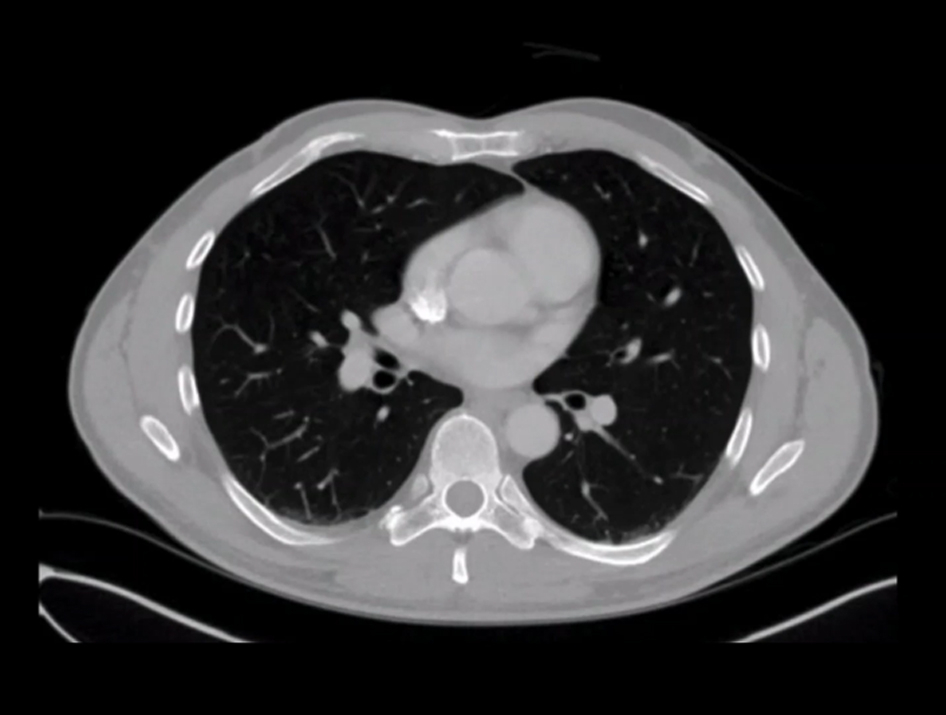

Supplement: Video 1 — Preoperative axial and sagittal computed tomography images demonstrating the location of the tumor. Video available at: https://www.jtcvs.org/article/S2666-2507(23)00108-6/fulltext. [file fx2.jpg]

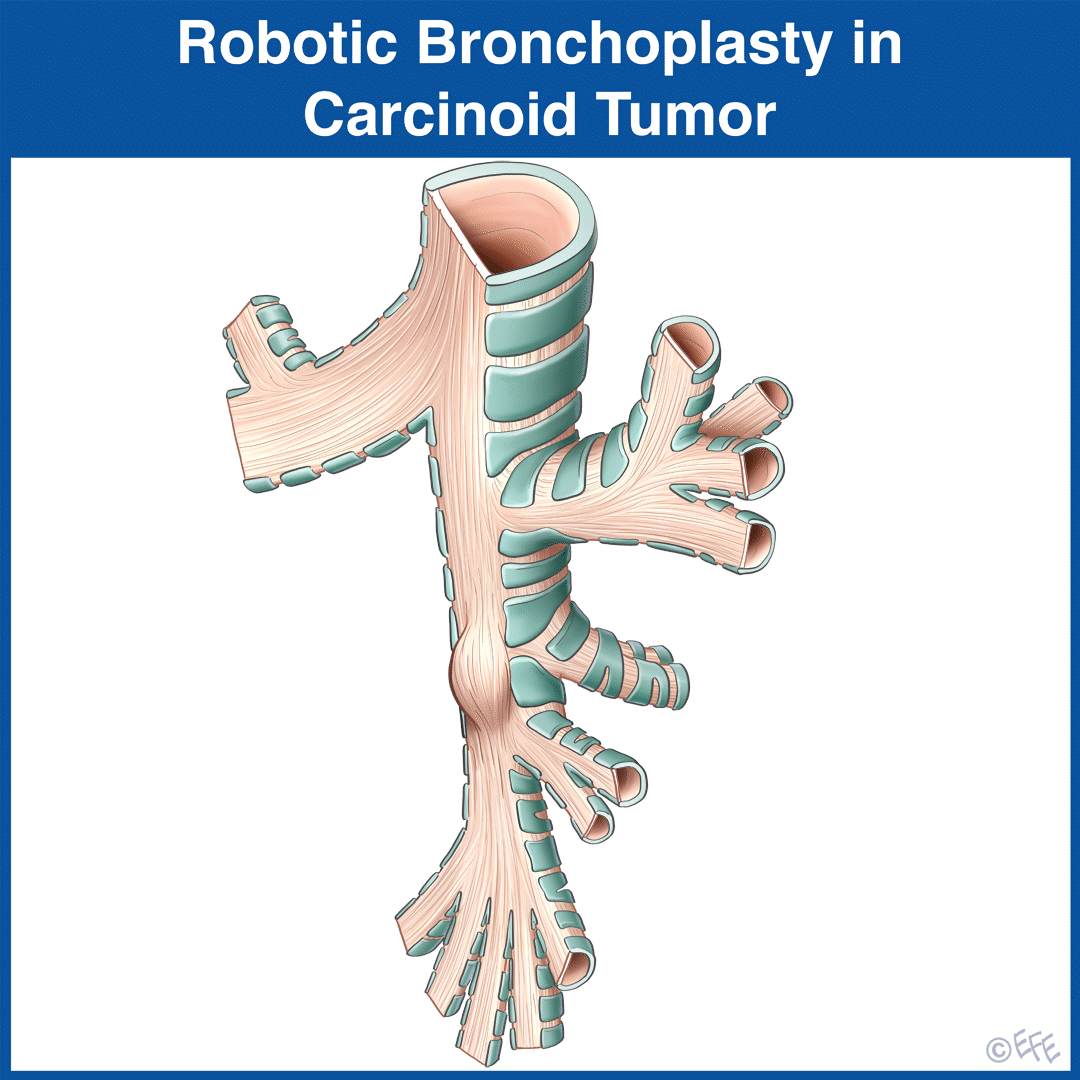

Supplement: Video 2 — Animation of plasty technique used for resection and reconstruction. Video available at: https://www.jtcvs.org/article/S2666-2507(23)00108-6/fulltext. [file mmc2.gif]

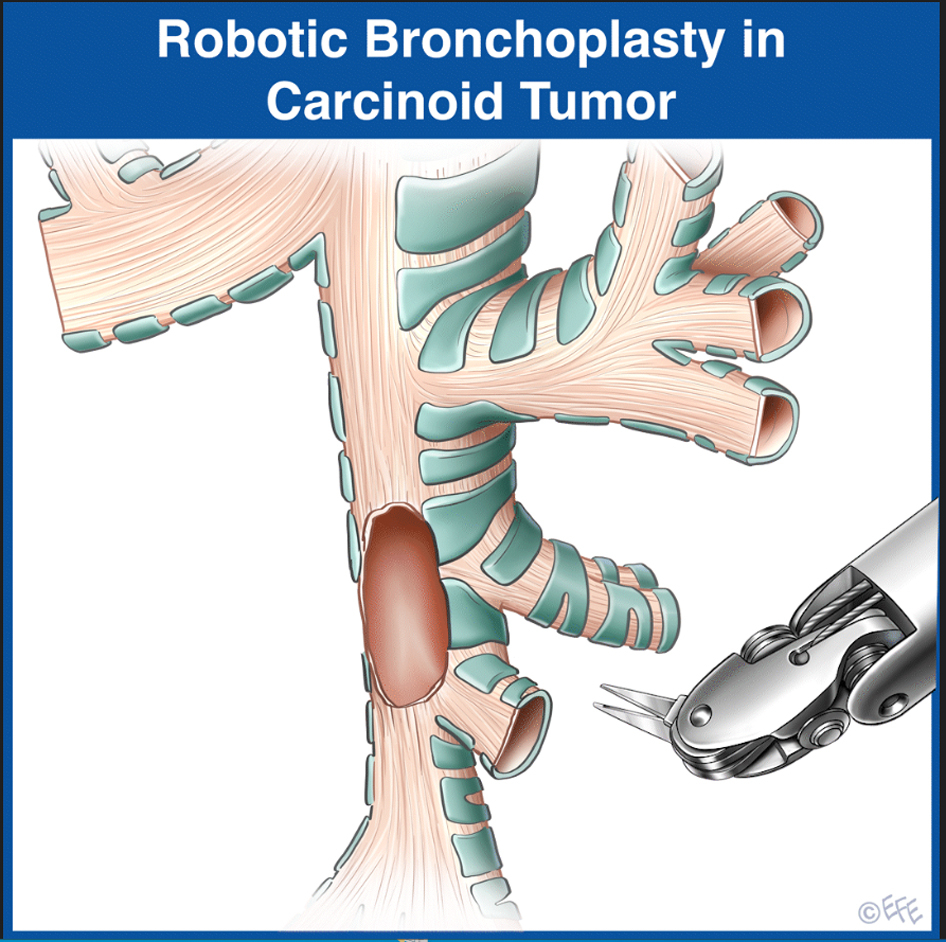

Supplement: Video 2 — Animation of plasty technique used for resection and reconstruction. Video available at: https://www.jtcvs.org/article/S2666-2507(23)00108-6/fulltext. [file fx3.jpg]

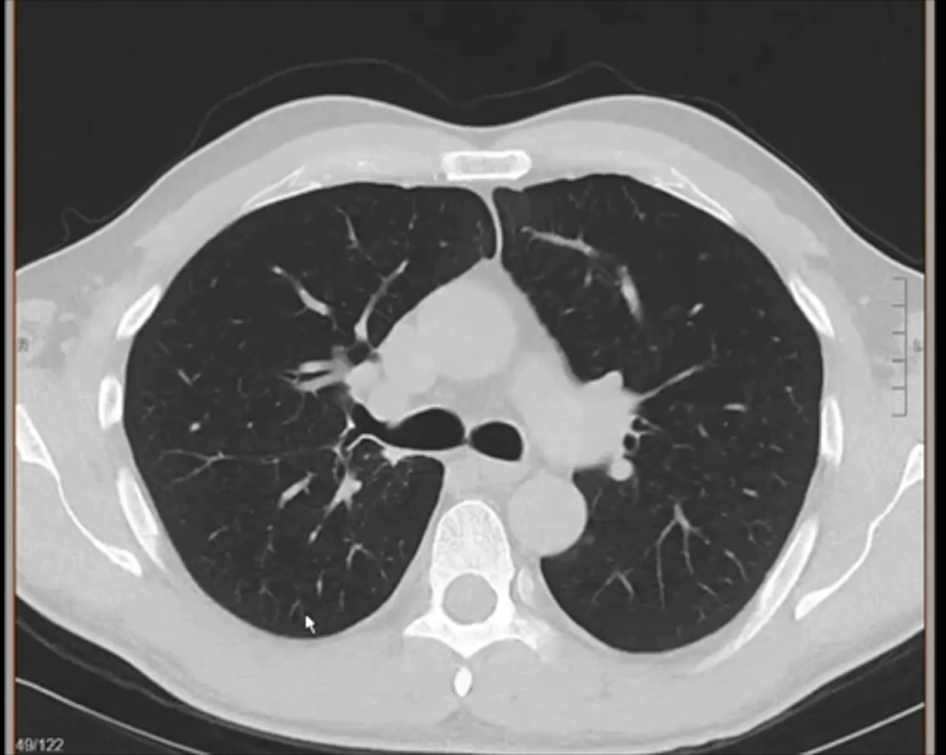

Supplement: Video 3 — Postoperative surveillance axial computed tomography images demonstrating widely patent reconstruction and pericardial fat pad-thymic flap coursing through fissure. Video available at: https://www.jtcvs.org/article/S2666-2507(23)00108-6/fulltext. [file fx4.jpg]

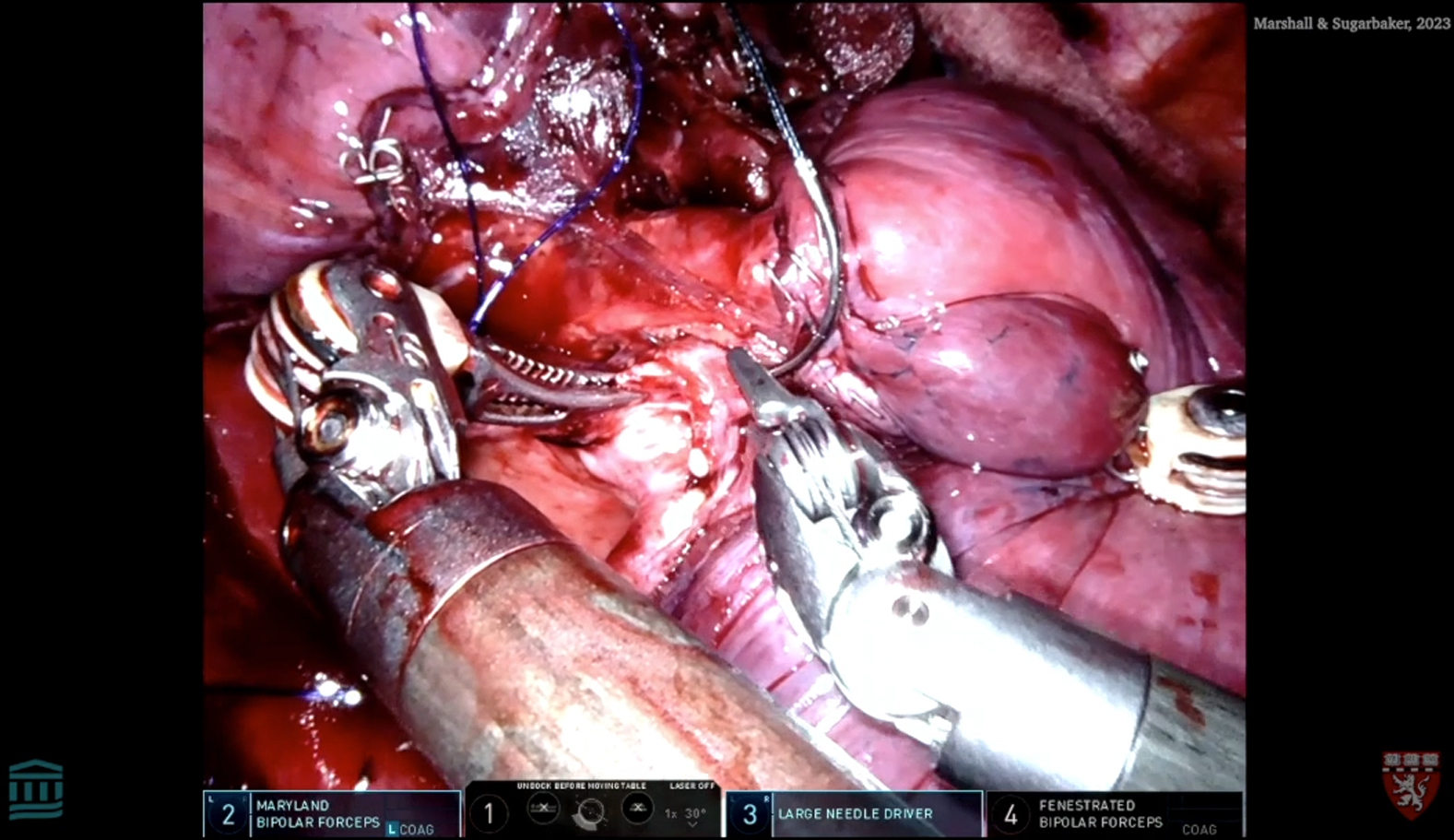

Supplement: Video 4 — Comprehensive case video with voice over corresponding description. Video available at: https://www.jtcvs.org/article/S2666-2507(23)00108-6/fulltext. [file fx5.jpg]
